# Implication and challenges of direct-fed microbial supplementation to improve ruminant production and health

**DOI:** 10.1186/s40104-021-00630-x

**Published:** 2021-10-12

**Authors:** Yajing Ban, Le Luo Guan

**Affiliations:** Department of Agricultural, Food and Nutritional Science, University of Alberta, Edmonton, Alberta T6G 2P5 Canada

**Keywords:** DFM-microbial interaction, Direct-fed microbials, Gut health, Host-DFM interaction, Ruminants

## Abstract

Direct-fed microbials (DFMs) are feed additives containing live naturally existing microbes that can benefit animals’ health and production performance. Due to the banned or strictly limited prophylactic and growth promoting usage of antibiotics, DFMs have been considered as one of antimicrobial alternatives in livestock industry. Microorganisms used as DFMs for ruminants usually consist of bacteria including lactic acid producing bacteria, lactic acid utilizing bacteria and other bacterial groups, and fungi containing *Saccharomyces* and *Aspergillus*. To date, the available DFMs for ruminants have been largely based on their effects on improving the feed efficiency and ruminant productivity through enhancing the rumen function such as stabilizing ruminal pH, promoting ruminal fermentation and feed digestion. Recent research has shown emerging evidence that the DFMs may improve performance and health in young ruminants, however, these positive outcomes were not consistent among studies and the modes of action have not been clearly defined. This review summarizes the DFM studies conducted in ruminants in the last decade, aiming to provide the new knowledge on DFM supplementation strategies for various ruminant production stages, and to identify what are the potential barriers and challenges for current ruminant industry to adopt the DFMs. Overall literature research indicates that DFMs have the potential to mitigate ruminal acidosis, improve immune response and gut health, increase productivity (growth and milk production), and reduce methane emissions or fecal shedding of pathogens. More research is needed to explore the mode of action of specific DFMs in the gut of ruminants, and the optimal supplementation strategies to promote the development and efficiency of DFM products for ruminants.

## Introduction

With the increased demand for animal products and the public’s concerns on the negative consequences of the livestock production, the industry has faced the pressure to improve animal production and health, reduce the negative environmental impact, and enhance animal products’ safety. Currently, feed additives including antimicrobial growth promotors, enzymes, probiotics, prebiotics and so on have been supplemented to animal diets to achieve these goals. The use of antibiotics (e.g., avoparcin, tylosin and chlortetracycline for swine and poultry productions, tetracyclines and monensin for ruminant production) as growth promoters in animal feeds is a widely applied practice in livestock industry because antibiotics can improve overall health of livestock that can result in an increase in body weight (BW) gain and feed efficiency. However, it has evoked a great concern in public due to the potential risks of contamination in animal food products (milk or meat) with antibiotic residues, the development of antibiotic resistance in microbes associated with animal or human diseases, and the transmission of the antibiotic residues and antibiotic resistance to the environment [1, 2]. Given these and the banned or to-be-banned usage of antibiotics as growth promotants and/or prophylactic treatment across many countries like the European Union and North America, it is crucial to develop alternative strategies to replace antibiotic growth promoters in livestock industry.

The application of probiotics has become popular as one of the alternatives to antibiotics with the aim to maintain and improve livestock performance as well as animal health. The “probiotics” are defined by the Food and Agriculture Organization (FAO) and World Health Organization (WHO) as “live microorganisms which when administered in adequate amounts confer a health benefit on the host” [3]. The terms “probiotics” and “direct-fed microbials” are used interchangeably, but in practice they are not synonyms. Based on the US Food and Drug Administration, direct-fed microbials (DFMs) are defined as the feed products containing only a source of live naturally existing microbes [4], while probiotics can contain enzymes or crude extracts [5]. DFMs have been used in all livestock sectors including swine [6, 7], poultry [8, 9], cattle [10, 11], lamb [12, 13], and young ruminants [14, 15] in the past two decades. To date, the mode of action of DFMs in pigs and poultry have been widely studied with their effects to alter microbial ecology in the digestive tract and thus improve nutrient absorption and/or immune functions, and these outcomes have been reviewed extensively [16–18]. However, the research on DFMs in ruminants is not as extensive as in the monogastric animals, with the majority focused on the impact of DFM supplementation on the rumen fermentation and production performance [19, 20] but fewer have been conducted to evaluate their effects on the lower gastrointestinal tract (GIT). Additionally, there have been conflict findings in ruminant research, as some of the studies showed positive results [21, 22] of using DFMs while others reported no effect [23, 24]. Furthermore, some practical challenges of DFMs such as microbial characterization of DFMs, their viability *in vivo*, interactions with endogenous gut microbiota and the host have not been clearly defined in ruminants, preventing their applications in ruminant farming. Therefore, this review will focus on the recent studies on DFM application in ruminants. It is noticeable, the implication of DFM in ruminants has been reviewed by McAllister et al. [25] in 2011 with the proposed mechanistic actions of DFMs. Thus, the current review will critically review and summarize the DFMs that are available and studies performed in the last 10 years with the aim to discover what new knowledge has been generated and what are the key barriers and challenges for the next decade to apply DFMs in ruminant production.

### Direct-fed microbials for ruminants

#### Types of DFMs

The microorganisms that can be used as DFMs to target improving ruminant production and health have been studied for over 25 years. The following criteria are commonly used for the selection of microbial strains to be used as DFMs: (1) confer health benefits on the host animal [26]; (2) be able to adhere to and colonize the gut epithelia [27]; (3) be competitive with pathogens for colonization of mucosa and/or for nutrients in the gut, and can stimulate desired microbial fermentation [28, 29]; (4) can produce antimicrobial substances (e.g., organic acids, bacteriocins, hydrogen peroxide) and produce or stimulate enzyme secretion [29, 30]. As summarized in Table 1, many bacteria and fungi have been investigated and applied as DFMs for ruminants, and the commercial DFM products may consist of single- or multi-species of microbes. Among them, the majority of bacterial DFMs are classified as lactic acid producing or utilizing bacteria including species of *Lactobacillus*, *Bifidobacterium*, *Streptococcus*, *Enterococcus*, *Megasphaera* and *Propionibacterium*, which are microorganisms like DFMs used for human and monogastric animals [17, 53]. Moreover, fungal species of *Saccharomyces* and *Aspergillus* are applied as DFMs in ruminant diets. In the following section, the proposed modes of action are discussed for different DFM sources.

**Table 1:** Microorganisms used as direct-fed microbials (DFMs) for ruminants

Microorganism (Genus)	Species	References
**Lactic acid producing bacteria**		
*Lactobacillus*	*L. acidophilus*	[10, 31]
	*L. casei*	[32, 33]
	*L. gallinarum*	[29]
	*L. plantarum*	[15, 34]
	*L. reuteri*	[35, 36]
	*L. bulgaricus*	[29]
	*L. delbrueckii*	[37]
	*L. rhamnosus*	[20]
*Bifidobacterium*	*B. pseudolongum*	[29]
	*B. thermophilium*	[29]
	*B. longum*	[38, 39]
	*B. lactis*	[29]
	*B. animalis*	[38]
*Streptococcus*	*S. bovis*	[29]
	*S. faecium*	[29]
*Enterococcus*	*E. faecium*	[10, 40]
	*E. faecalis*	[29]
**Lactic acid utilizing bacteria**		
*Megasphaera*	*M. elsdenii*	[41, 42]
*Propionibacterium*	*P. shermanii*	[29]
	*P. freudenreichii*	[29]
	*P. acidipropionici*	[43]
	*P. jensenii*	[43]
**Other bacteria**		
*Prevotella*	*P. bryantii*	[44]
*Bacillus*	*B. subtilis*	[45, 46]
	*B. amyloliquefaciens*	[47]
	*B. toyonensis*	[48]
	*B. licheniformis*	[49]
	*B. coagulans*	[50]
**Yeast**		
*Saccharomyces*	*S. cerevisiae*	[11, 51]
	*S. boulardii*	[14]
**Other Fungi**		
*Aspergillus*	*A. oryzae*	[52]

#### Proposed mode of action in the rumen

**Fig. 1:**
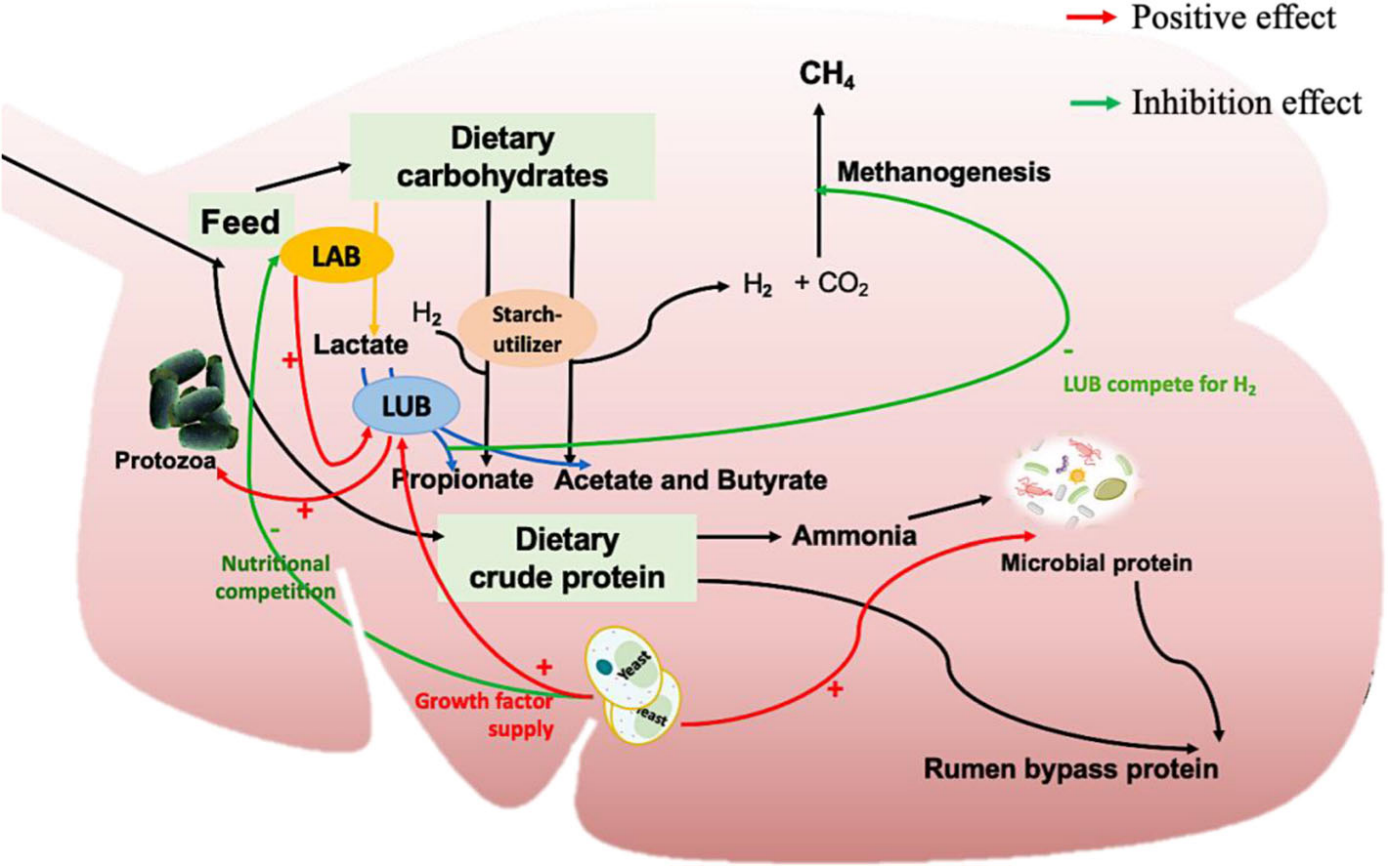
Mode of action of proposed DFMs in the rumen. The roles of lactic acid producing bacteria (LAB), lactic acid utilizing bacteria (LUB), other bacteria that can utilize starch (starch-utilizer), and yeast in rumen fermentation are shown in respect of carbohydrate fermentation and microbial protein synthesis

For ruminants, since rumen is the first site for the inoculation, ruminal microbial ecology, microbial fermentation, and microbial protein synthesis can be affected by DFMs (Fig. 1). As lactic acid production and utilization in the rumen is closely associated with rumen health and feed digestive efficiency [54, 55], DFMs which can produce or utilize lactate have been demonstrated to beneficially affect rumen fermentation and prevent ruminal acidosis [11, 20].

##### Lactic acid producing and utilizing bacterial DFMs

Ruminal acidosis is a main metabolic disease in ruminants when animals ingest large amounts of highly fermentable carbohydrates (i.e., starch) or their diet is rapidly changed from a high forage-based diet to a high-concentrate type [56]. When ruminal pH falls below 5.5, high concentrations of total volatile fatty acids (VFA) or lactic acid can be accumulated in the rumen, leading to subacute or acute acidosis [57]. In the rumen, the main lactic acid producing bacteria (LAB) are *S. bovis*, *Lactobacillus spp.* and *S. ruminantium* which can proliferate rapidly under low-pH conditions [58]. The overgrowth of *S. bovis* is associated with mild subacute ruminal acidosis (SARA), and the abundance of *E. coli* was associated with severe SARA and inflammation [59]. Therefore, LAB such as *Lactobacillus* and *Enterococcus* have been proposed as DFMs due to their potentials in boosting the ruminal microbes adapting to the presence of lactic acid in the rumen [5] and stimulating lactic acid utilizing bacteria (LUB) [29]. In addition to these proposed mechanisms, we speculate that these LAB can compete with *S. bovis* and their produced bacteriocins could inhibit the *E. coli* growth in the rumen as shown in *in vitro* studies [53, 60]. However, such studies did not test whether these LAB can inhibit these potential ruminal acidosis associated bacteria *in vivo*.

In the rumen*,* LUB such as *S. ruminantium* and *M. elsdenii* can utilize lactic acid to produce VFA [29], and thus LUB-based DFMs are expected to aid in preventing the accumulation of lactate in the rumen and maintaining a higher ruminal pH as well as increasing VFA production. *M. elsdenii* is one of the species to prevent ruminal acidosis in dairy cows fed a high-concentrate diet, especially for the postpartum period [41, 61]. This species seems to be the major lactic acid utilizer in the rumen when animals are fed readily fermentable carbohydrates, since other lactate-fermenting bacteria (*S. ruminantium*) undergo catabolite repression [62, 63]. Furthermore, it has been reported that *M. elsdenii* utilizes lactate, glucose and maltose thus would compete with LAB for substrate, leading to reducing the lactate concentration and in the meantime enhancing VFA production [41, 64]. It is also suggested that the efficacy of *M. elsdenii* on prevention of SARA might depend on the dietary grain types and the endogenous rumen microbiota, and meanwhile, the supplementation of this bacterial species can alter the rumen microflora by decreasing *S. bovis* counts and increasing protozoa counts [42]. However, the past research did not take the individualized rumen microbiome into account on how they respond to this DFM differently. Such limitations could be the barrier of applying *M. elsdenii* as DFMs in different production systems.

*Propionibacterium* is another potential LUB DFM for its function on utilizing lactate to produce propionate, the major precursor for gluconeogenesis in the liver of ruminants [65]. Supplementation of *Propionibacteria* has been reported to provide more substrate for lactose synthesis, improve energetic efficiency and potentially reduce ketosis in early lactation dairy cows [65]. In addition, increased propionate may reduce hydrogen available for enteric methane production in the rumen [66]. However, supplementation of *Propionibacterium spp.* failed to affect total VFA production or enteric methane production in beef heifers fed high-forage diets [43, 67] and finishing cattle fed high-concentrate diets [68]. These studies observed unchanged propionate in the rumen of finishing cattle due to the moderate persistency of the inoculated strains within the rumen microbiome and/or the pre-existing high propionate production from high starch fermentation [43, 68]. Overall, *Propionibacterium* may exert its actions differently in cattle fed barley- or corn-based diets, suggesting that the mode of action of *Propionibacterium* supplementation can be diet-dependent.

##### Other bacterial DFMs

Some rumen bacteria (e.g., *P. bryantii*) have been considered as potential DFMs because they can utilize starch to produce succinate and propionate [69]. *Prevotella bryanti* has shown to reduce rumen lactate concentration and increase ruminal fermentation products (e.g., acetate, butyrate), however, it could not effectively regulate ruminal pH and prevent SARA [44]. In addition, several species of *Bacillus* (e.g., *B. subtilis*) are currently used as DFM supplements with their ability to improve the degradation of dietary carbohydrate and protein, increase populations of amylolytic and proteolytic bacteria in the rumen, promote the proliferation of *Lactobacilli*, modify rumen fermentation, and increase total VFA concentration [45, 70]. However, past studies only dealt with the compositional changes of rumen microbiota and fermentation products affected by *Bacillus* supplementation in dairy cows with limited information on its effect on active rumen microbiota and functions. Further research is needed to investigate the role of administered *Bacillus* in regulating rumen microbiome at both compositional and functional levels and in different ruminant species.

##### Fungal DFMs

Fungal DFMs especially yeast products (e.g., *S. cerevisiae, S. boulardii*) have been intensively used in ruminant industry. Their modes of action in the rumen [40, 71–75] can be summarized as: (1) stimulation of rumen microbial growth; (2) increasing fiber degradation and microbial protein flow to the small intestine; (3) altering rumen fermentation and VFA production.

Supplementation of *S. cerevisiae* has been reported to increase dry matter intake (DMI), rumen pH, VFA, and decrease ruminal lactate concentration in dairy cows [11, 40]. Although *S. cerevisiae* can metabolize lactate, its direct effect on altering ruminal lactate concentrations is minimal [25]. In addition to its potential function in oxygen scavenging [71], *S. cerevisiae* has been proved to promote the growth of cellulolytic microbes (e.g., *F. succinogenes*, *R. albus*), LUB (e.g., *S. ruminantium*), and amylolytic bacteria (e.g., *Ruminobacter*, *Bifidobacterium*), potentially leading to higher VFA concentration in the rumen after the supplementation [72, 73]. This promotion effect may be explained by the fact that *S. cerevisiae* may provide growth factors, such as organic acids, vitamins, or amino acids, to stimulate ruminal bacterial populations [74]. The supplementing *S. cerevisiae* could also lead to improved microbial nitrogen metabolism thus increasing microbial protein flow to the intestine and reducing N loss [75]. Alternatively, the competition with LAB for available sugar can contribute to the potential of SARA alleviation [74]. However, the persistence and viability of live yeast cells are reported to be low in the rumen since certain strains cannot colonize the rumen for a long period of time [76]. Thus, selecting strains which have a good survival in the rumen as yeast DFMs is vital to improve the efficacy of supplementation.

*Aspergillus oryzae* and *Aspergillus niger* are two fungal species used in animal diets commonly in the form of culture, culture extract or crude enzyme extracts [52, 77–79]. The previous reviews all considered them as DFMs because these crude extract products contain live cells [5, 29, 80]. However, based on the current definition that the crude culture extract or enzyme extracts should not be considered as true DFMs, this review only introduces the *Aspergillus oryzae*. Sun et al. [52] reported the effect of *A. oryzae* culture on rumen fermentation *in vitro* based on the observation of the stimulated growth of ruminal fungi (e.g., *Neocallimastix frontalis* EB 188) and cellulolytic microbes (e.g., *F. succinogenes*, *R. flavefaciens*). To date, little information is available about the mechanisms of live *A. oryzae* supplementation in the rumen of ruminant species.

#### Proposed mode of action in the lower gut

**Fig. 2:**
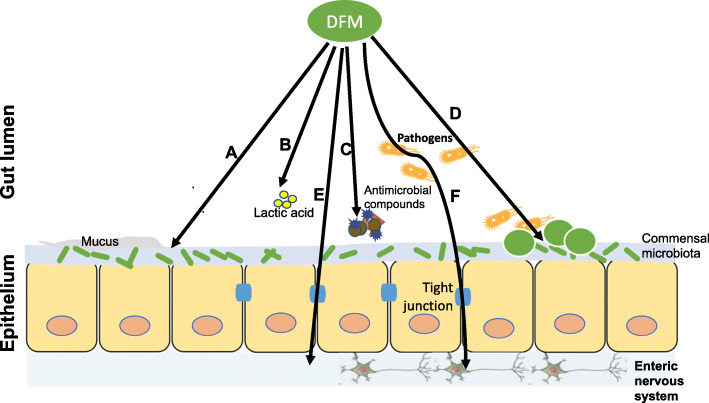
Mode of action of proposed DFMs in the lower digestive tract of ruminants. **A** Enhancement of gut barrier function; **B** Reduction of luminal pH by producing lactic acid; **C** Production of antimicrobial compounds (e.g., organic acids, hydrogen peroxide, bacteriocins); **D** Adherence and competing for nutrients; **E** Stimulation of host immune response; **F** Stimulation of expression and turnover of neurotransmitters (e.g., serotonin)

Although the modes of action of DFMs in the lower gut have not been well defined in ruminants, it is generally accepted that they can improve microbial balance, decrease the concentration of pathogens, affect host nutrient absorption and immune responses in the lower gut as defined in the monogastric animals [8]. The main proposed antagonistic roles of DFMs (Fig. 2) related to immune function and manipulation of gut microbial ecosystem are [57, 81–85]: (1) lowering the pH in the gut to inhibit pathogens’ growth; (2) competing with pathogens for adhesion sites and/or for nutrients; (3) secreting antimicrobial compounds; (4) enhancing gut barrier function; (5) stimulating host immune responses; and/or (6) modulating gut-brain communication and thereby reducing stress and improve animal behavior.

##### Modulation of microbial composition and pathogen colonization

Previous research has revealed that the DFMs can alter the gut microbial composition to increase beneficial bacteria (e.g., *Fecalibacterium*) and suppress opportunistic pathogens [14, 81]. Effective bacterial DFMs should have the ability to attach to the intestinal wall and compete with pathogens for sites of adherence and colonization [82]. Lactic acid producing bacteria have demonstrated inhibitory activities against pathogens by producing antimicrobial compounds, including organic acids (e.g., lactate, acetate), hydrogen peroxide (H_2_O_2_), and bacteriocins [83]. In addition to the lactic acid, the H_2_O_2_ produced by *L. lactis* can oxidize sulfhydryl groups of proteins and membrane lipids and inhibit pathogenic bacteria without catalase [86], however, such inhibition effectiveness in the gut is questionable because oxygen is needed [87]. In addition, bacteriocins produced by LAB can inhibit closely related strains and a wider range of bacterial species. McAllister et al. [25] reviewed previous research on bacteriocins produced by rumen bacteria and suggested that bacteriocin production is one of the key modes of action of most bacterial DFMs. However, such information has not been well studied in the lower gut of ruminants. Recently studies have reported the Lactobacilli colonizing in the gut of calves during early life [35, 88], whether the bacteriocins can be produced by these LAB warrants further research for their potentials as DFMs to inhibit pathogen colonization.

##### Enhancing host immunity and gut barrier function

Another proposed mechanism by which DFMs can improve animal performance is their impact on intestinal structure thickness. Lesions caused by enteropathogens in the lower gut of ruminants induce inflammation and consequently may lead to a thicker intestinal wall [89]. Elam et al. [90] found that the lumina propria was thinner at ileal region of steers fed DFMs compared to control steers, suggesting that more energy expenditure for growth with reduced inflammation by DFMs. Bacterial DFMs may also induce host immunomodulation via the mechanisms involving regulation of gene expression and signalling pathways in host immune cells [91]. The inoculated DFMs can be taken up by the intestinal epithelial cells via transcytosis, and subsequently macrophages or dendritic cells may engulf them to stimulate immune response. Krehbiel et al. [57] summarized bacterial DFM studies on host immunity in human and livestock, concluding that bacterial DFMs have the potential to affect cytokine production and T and B cell responses, depending on the strains, dose and feeding strategies of DFMs as well as host tissue types. Raabis et al. [91] recently reviewed probiotic studies using different animal and cell models and indicated that identified candidate genes on the genome of DFM strains can involve in bacteriocin secretion and modulation of host cytokine response. Additionally, they stated that the immunomodulatory effects of DFM strains may vary in different gastrointestinal regions in the host. To date, many studies on DFMs and their roles in host immune functions have been conducted in human and monogastric animals, however, fewer studies have reported their potentials in induced immune responses in ruminant species. Although innate immune responses (e.g., increased phagocytosis and natural killer cell activity) and increased immunoglobulin A (IgA) as well as decreased immunoglobulin E have been observed in human [92], supplementing different strains of *L. acidophilus* and *P. freudenreichii* showed no effect on the serum IgA concentrations in feedlot cattle [90]. Also, administration of *Lactobacillus spp.* to neonatal calves did not change cytokine expression and IgA levels as well [36]. Further research is needed to target the specific immune responses induced by inoculation of different species of DFMs on ruminant species to find effective supplementation strategies for gut health.

##### Alteration of microbial metabolites affecting animal behavior

More recently, attention has been attracted on the role of DFMs on animal behavior by means of the microbiota-gut-brain axis that gut microbiota derived metabolites can communicate with the central nervous system and brain via neurons, endocrine, and immune mediators [93]. With a large and growing body of studies investigating the bidirectional communication and interaction of gut microbiota and nervous systems (enteric nervous system and central nervous system) using germ-free rodent models, it has been demonstrated that bacterial colonization of the gut is central to the development of both enteric and central nervous systems [94]. Gut bacteria can produce neurotransmitters (e.g., γ-aminobutyric acid (GABA), serotonin), which may induce gut epithelial cells to secrete molecules to modulate neural signaling within the enteric nervous system and thus influence brain function and host behavior [84]. In addition, Kraimi et al. [85] indicated that DFM fed to the farm animals can modify gut microbial composition and release of microbial metabolites (e.g., short-chain fatty acids, neurotransmitters, catecholamines), and consequently may affect the anxiety-like behavior, memory capacities, social behavior, and feeding behavior of monogastric animals. However, such effect of DFMs has been reported for feeding behavior in ruminants supplemented with yeast *S. cerevisiae* [95].

The effect of yeast supplementation is generally considered in the rumen, but yeast cells can remain alive though transition to the lower digestive tract [96]. The mode of action of yeast in the lower gut of ruminants has not been fully elucidated, and there is no clear evidence showing the beneficial effect of yeast on the feed digestion in lower GIT. Given its functions of enhancing microbial protein synthesis and cellulolytic bacteria in the rumen, it may have the potential to improve protein and carbohydrate digestion in the lower digestive tract of ruminants, but the mechanisms should be further studied. In general, the bacterial and fungal DFMs are considered to have different modes of action in the rumen and lower gut of ruminants. Further studies are needed to specifically focus on the effect of DFMs on the lower digestive tract of ruminant species.

### Effect of DFMs on ruminant health and performance

DFMs have been considered as potential alternatives to antibiotics for neonatal and young ruminants, transition and lactating dairy cows, receiving and feedlot beef cattle, and small ruminants for the purpose to improve animal health, daily gain, milk production, and/or feed efficiency. Numerous studies have been focused on the impact of single- or multi-strain DFMs in animal health and performance. To evaluate their effects on animal health or immune responses, pathogen challenge (inoculation with live pathogens) and natural challenge (natural exposure to pathogens in an environment similar to practical production settings or stress challenge) models have been used. In this section, health and performance related responses to various DFM supplementation (e.g., divergent types of DFMs, dosage levels, delivery methods) in different animal growth and production stages are discussed based on studies performed in the past 10 years.

#### Effect of DFMs on pre-weaning or weaning ruminants

Most of the current research on the efficacy of DFMs pays particular attention to neonatal and pre-weaned ruminants whose gut structure and functions are similar to those of monogastric animals. Especially, their immune functions are not fully developed, and they are under highly stressed conditions due to environmental and nutritional changes, dehorning, vaccination, and weaning. As a result, the chance of colonization by pathogenic bacteria increases [97] and the protective potential of the beneficial gut microbiota decreases [98]. Additionally, as the rumen is not developed and functional, young ruminants have to digest feed in the lower gut, therefore, the risk of intestinal proliferation of harmful microorganisms is higher compared to adult animals [29]. The supplementation of DFMs is expected to aid young animals to establish and maintain the intestinal microbiota, prevent dysbiosis, and restore gut functions [99]. Therefore, divergent strains and dosages of DFMs have been tested on young ruminants in different life stages from neonate to post-weaning (Table 2).

**Table 2:** Impact of different DFM supplementation on pre-weaning or weaning young ruminants’ health and performance

DFM	Dosage	Delivery method	Animal	Health	Performance	Reference
*L. plantarum* GB LP-1	4 g: 4.8 × 10^9^ cfu/d 8 g: 9.6 × 10^9^ cfu/d	In milk replacer	Neonatal dairy calves	Fecal scores improved with increasing inclusion rates	Increased weight gain and feed efficiency; similar starter intake; greatest feed efficiency at 4 g/d	[15]
*L. casei* DSPV 318 T *L. salivarius* DSPV 315 T *P. acidilactici* DSPV 006 T	3 × 10^9^ cfu/kg BW	Suspended in 0.15 mol/L NaCl	Pre-weaning dairy calves	Lower fecal consistency index	Higher ADG, starter intake; earlier consumption of starter and earlier development of the rumen	[32]
*L. animalis SB310* *L. paracasei SB137* *B. coagulans SB117*	30:35:35: 1.8 × 10^10^ cfu/d	In milk replacer	First-month dairy calves	Lower incidence of diarrhea	Improved BW, total concentrate intake, heart girth	[50]
*B. subtilis*	3 × 10^9^ cfu/dose	In electrolyte	Pre-weaning scouring dairy calves	Promoted T cell subsets, alleviated inflammation	–	[100]
*S. cerevisiae boulardii*	0.5 g/d	In milk and/or grain	Neonatal dairy calves with failure of passive transfer	Supplementation in grain: decreased days in diarrhea	Supplementation in grain: more starter intake and faster growth prior to weaning	[101]
*S. cerevisiae boulardii* CNCM I-1079	10 × 10^9^ cfu/d	In milk replacer	Pre-weaning dairy calves	Alleviated diarrhea and maintained a health bacterial community with *Fecalibacterium* as the predominant genus	No effect on feed intake; similar ADG between diarrheic calves fed with yeast and nondiarrheic calves	[14]
*S. cerevisiae boulardii*	1 × 10^10^ cfu/d	In milk replacer	Pre-weaning dairy calves	No effect on health scores, fecal biomarkers of gut health	No effect on intake, metabolizable energy intake, ADG, feed efficiency	[102]
*C. tropicalis*	5 × 10^9^ cfu/d	In the basal diet	Preweaning dairy calves with *E. coli* K99 challenge	Lower copy numbers of *E. coli* K99 in jejunum digesta; reduced days of diarrhea	No effect on ADG and DMI	[103]
*D. hansenii* CBS 8339	0.7 g/kg BW/d	In milk	Newborn goats with *E. coli* challenge	Enhanced respiratory burst, catalase activity, superoxide dismutase activity after challenge at d 15; increased peroxidase activity, nitric oxide production, catalase activity after challenge at d 30; upregulated expression of genes *TLR* (2, 4, 6), modulator genes *Raf.1*, *Syk* and *Myd88*, transcription factor gene *AP-1*, and cytokine genes *IL-1β* and *TNF-α* at d 15	–	[104]
*S. cerevisiae boulardii* *or* *L. acidophilus*	SCB: 7.5 × 10^8^ cfu/L milk replacer + 3 × 10^9^ cfu/kg starter; LA: .5 × 10^8^ cfu/L milk replacer + 1 × 10^9^ cfu/kg starter	In milk replacer and starter feed	Weaning dairy calves	SCB and LA reduced potential pathogenic *Streptococcus* and *Tyzzerella_4*, increased beneficial bacteria	–	[105]
*B. subtilis*	13 g/d	In starter ration	Weaned Holstein steers with *Salmonella* challenge	Reduced *Salmonella* concentrations in jejunum, ileum, colon at 48 h post-challenge but no difference at 96 h after challenge; increased white blood cells and lymphocyte counts	Greater feed intake before and after challenge	[46]
*M. elsdenii* NCIMB 41125	5 × 10^9^ cfu/d	25 mL suspension	Pre- and post-weaning	–	No effect on rumen fermentation, blood metabolites associated with butyrate	[106]

*ADG* Average daily gain, *BW* Body weight, *DMI* Dry matter intake

High mortality and morbidity often occur in the neonatal and young ruminants, which is usually associated with diarrhea. There is a widely established view that LAB, primarily *Lactobacillus*, could suppress diarrhea and improve growth of young or stressed calves. Supplementation of a strain of *L. plantarum* (GB LP-1) to neonatal dairy calves with two different doses (4 or 8 g/d, in milk replacer feeding) improved BW gain and feed efficiency as well as improving fecal scores with higher dosage supplementation [15]. Similarly, pre-weaning calves fed a combination of *Lactobacillus* and *Pediococcus* had higher average daily gain (ADG), starter intake and lower fecal consistency index [32]. Another study also reported that supplementation of the combination of *Lactobacillus* and *Bacillus* reduced incidence of diarrhea, increased concentrate intake and growth (BW, heart girth) in calves during their first month of life [50]. With the ability to produce acetate and lactate and form biofilm [107], another LAB, bifidobacteria, have been isolated from calves and tested as DFM candidates for reducing diarrhea in calves [38], or supplemented with other bacterial species [108]. However, due to the proposed low bifidobacteria survival rate in calves [39], further studies are required to discover suitable strains or strains from different sources for bifidobacteria-based DFMs with better persistence. Differently from bifidobacteria, the spore-forming *Bacillus spp*. are regarded to have better viability in the GIT. Scouring calves orally supplemented with electrolyte containing *Bacillus subtili* (3 × 10^9^ cfu/dose) showed enhanced immunity (greater proportions of CD8 − CD25^+^, CD8 − CD45RO^+^, CD8 − TCR1^+^) and alleviated inflammation compared to scouring calves treated with electrolyte alone and non-scouring calves [100]. Besides, live yeast products have been studied intensively in dairy calves and show their benefits on calf health and growth. A previous study has shown that *S. cerevisiae boulardii* (0.5 g/d) can promote starter intake and growth of neonatal Holstein calves with failure of passive transfer and in the meantime alleviating diarrhea when live yeast was added to the grain feeding [101]. A recent study showed *S. cerevisiae boulardii* CNCM I-1079 maintained ADG in scouring preweaning calves compared with non-diarrheic calves [14]. Another fungal species *C. tropicalis* added to the basal diet was shown to reduce days in diarrhea of preweaning calves with *E. coli* K99 challenge, but ADG and dry matter intake (DMI) of the calves were not changed [103]. These outcomes from above animal trials suggest that DFM supplementation to neonatal calves can benefit intestinal gastrointestinal tract and promote intestinal health and host immunity as well as body weight gain in pre-weaning calves.

Small ruminants play an important role in small farm systems and agriculture economy in many European, Asian, Oceanian countries due to the demand of milk and meat from goat and sheep. To date, most of the studies have focused on growing and lactating small ruminants with few studies reported the beneficial effects of DFMs on these neonates at pre-weaning period. A recent study supplied a marine yeast strain *D. hansenii* CBS 8339 directly to newborn goats challenged with *E. coli* and found no effective protection against *E. coli* [104]. However, the yeast administration improved respiratory burst, catalase activity and superoxide dismutase activity, and upregulated the expression of *TLR* (*2, 4, 6*), modulator genes *Raf-1*, *Syk* and *MyD88*, transcription factor gene *AP-1*, cytokine genes *IL-1β* and *TNF-α* at d 15. This suggests a short effective time (15 days) of supplementing *D. hansenii* CBS 8339 to neonatal goats on promoting innate immune and antioxidant responses. However, more research is needed to study the potential DFMs to benefit the growth and health of neonatal small ruminants.

Weaning is another challenging and important period for young pre-ruminants, therefore, proper growth, rumen fermentation and gut health are the main goals for a smooth and less stressed transition. DFMs could stimulate host immune responses (increased white blood cells and lymphocyte counts) and promote starter intake as well as growth in weaning young ruminants. The supplementation of *S. cerevisiae boulardii* CNCM I-1079 (SCB) and *L. acidophilus* BT1386 (LA), respectively, to dairy calves during weaning showed that both SCB and LA reduced potential pathogenic *Streptococcus* (in colon mucosa) and *Tyzzerella_4* (in ileum mucosa) before weaning and increased beneficial bacteria such as *Fibrobacter* (in ileum mucosa) [105]. Similarly, *B. subtilis* supplementation showed decreased *Salmonella* concentrations in the gut digesta of weaned calves with *Salmonella* challenge and increase their feed intake [46]. However, past studies showed that the DFM supplementation is non-effective to the calves who have healthy conditions during pre-weaning to weaning transition [36, 102, 109]. Overall, these studies highlight the positive outcomes of DFM supplementation to weaning calves under the circumstance of health challenge. However, future studies are needed to take ages, exposure to environmental microorganisms, diets, management, or endogenous gut microbiome into consideration to deliver more solid outcomes of DFMs for young ruminants.

#### Effect of DFMs on growing or finishing ruminants

Transport, fasting, castration, transition to high-grain diets, regrouping and overcrowding are the potential stressors to growing ruminants when entering the feedlot operations and afterwards, which usually lead to decreased growth and increased morbidity and mortality. In addition, bovine respiratory disease (BRD) is one of the most challenging diseases in the North America beef cattle industry of which the estimated annual economic loss is approximately $600 million with the majority cases happening in the receiving period (normally first 28 d) at feedlots [81]. Feedlot ruminants face the transition of diets (from high-forage to high-grain) and new environment (from grazing to group-raising) when moved to the intensive farming operations. DFMs have been studied to improve growth performance, carcass traits, health of ruminants in the growing and finishing stages, and to reduce shedding of pathogens (Table 3).

**Table 3:** Impact of various DFM supplementation on health and performance of growing or finishing ruminants

DFM	Dosage	Delivery method	Animal	Health	Performance	Reference
*S. cerevisiae*	5 g/d	In ration	Receiving beef cattle with LPS challenge	No effect on leak cortisol concentrations	Improved cumulative DMI	[110]
*S. cerevisiae* CNCM I-1077	8 × 10^9^ cfu/d	Pelleted and mixed in rations with monensin	Receiving and backgrounding beef cattle	–	Increased BW, ADG, G:F on d 47; no effect on cumulative ADG, DMI, G:F during d 1–77	[111]
*L. acidophilus* *E. faecium*	10^9^ cfu/d	Top-dressed to the ration	Receiving beef cattle	No effect on morbidity, humoral immune response	No effect on DMI, feed efficiency; maintained fecal pH between d 7 and 14; performance response may relate to degradable intake protein levels	[10]
*S. cerevisiae*	Low: 3 × 10^10^ cfu/d High: 6 × 10^10^ cfu/d	In steam-flaked corn-based diets	Finishing beef cattle	–	No impact on DMI, ADG, G:F, feeding behavior; increased carcass quality with increasing yeast inclusion; increased total tract apparent digestibility of DM, OM, CP, EE, fiber	[51]
*S. cerevisiae*	1.7 × 10^10^ cfu/g	In ruminally protected and/or nonprotected forms and top-dressed to the diets	Finishing beef cattle	Higher LPS binding protein concentration; reduced liver abscess	No effect on DMI, final BW, ADG, G:F, carcass traits, NEFA; tended to reduce fecal shedding of *E. coli* in encapsulated yeast treated cattle	[112]
*S. cerevisiae* Sc47 CNCM I-4407	2.5, 5, or 10 g: 1 × 10^10^ cfu/g	In ration	Finishing beef cattle	–	Dose effect depended on the type of diet; affected digestibility of DM, fiber	[113]
*S. cerevisiae*	2 or 4 g/d: 2 × 10^10^ cfu/g	In ration	Finishing beef cattle	–	Improved ADG and ruminal propionate concentration; supplementation at 4 g/d shifted rumen microbial composition	[114]
*S. cerevisiae*	1.5 g/d	Top-dressed to the ration	Finishing beef cattle with heat stress	–	No effect on BW, ADG, water intake in thermoneutral conditions, complete blood counts, glucose, NEFA; water intake higher under heat stress, tendency in decreasing respiration rates under heat stress	[115]
*K. marximanus* NRRL3234 *S. cerevisiae NCDC42* *S. uvarum* ATCC9080	Single strain or mixed culture (1:1:1): 1.5–2.0 × 10^9^ live cells/kg BW	In ration	Feedlot lambs	–	No effect on intake, N intake and N in feces and urine; improved microbial CP synthesis and feed efficiency; NCDC42 and mixed DFM increased BW gain	[109]
*S. cerevisiae* *L. sporogenes*	1.5% of concentrate: SC: 1.3 × 10^11^ cfu/g; SC 1.5 × 10^11^ cfu/g and LS 5 × 10^10^ cfu/g	In ration	Weaned growing lambs	–	No effect on body weight, ADG, digestibility of all nutrients (except higher acid detergent fiber digestibility in treated groups); similar carcass traits	[12]
*S. cerevisiae*	5 × 10^8^ cfu/d	In ration	Finishing lambs	–	No effect on DMI, feed conversion, weight gain, rib eye dimensions, carcass subcutaneous fat thickness; increased carcass weight and length; increased rumination time	[116]
*L. acidophilus* NP51	10^9^ cfu/d	In ration	Finishing beef cattle	–	No effect on gain, intake, feed efficiency; reduced *E. coli* O158:H7 fecal shedding	[31]
*L. acidophilus* *L. casei*	1 × 10^9^ cfu/d	In ration	Finishing beef cattle	–	No effect on *E. coli* fecal prevalence or supershedding prevalence	[33]
*R. flavefaciens*	5.6 × 10^13^ powder/d; 1.1 × 10^14^ liquid/d	Powder or liquid form in concentrate feed mixture	Growing lambs	–	Increased digestibility, N utilization, total VFA, rumen volume, microbial N synthesis, gas production, and ADG; decreased ammonia, acetate, *in vitro* methane concentrations, protozoa count	[117]
*L. fermentum and L. plantarum* (FP) *S. cerevisiae (SC) plus FP* (SCFP) *M. elsdenii plus SCFP* (MSCFP)	FP: 4.5 × 10^8^ cfu/d; SCFP: 4.5 × 10^8^ cfu/d FP + 1.4 × 10^10^ cfu/d SC; MSCFP: 4.5 × 10^8^ cfu/d FP + 1.4 × 10^10^ cfu/d SC + 4.5 × 10^8^ cfu/d ME	Oral dosed with a 50 mL microbial suspension before morning feeding	Growing lambs	Higher populations of *R. albus* and *R. flavefaciens* in MSCFP and SCFP than control and FP; highest *M. elsdenii* and lowest methanogen abundance	No effect on feed intake, BW; increased ADG, feed efficiency in 21 d; highest protein supply in MSCFP lambs	[34]

*ADG* Average daily gain, *BW* Body weight, *CP* Crude protein, *DM* Dry matter, *DMI* Dry matter intake, *EE* Ether extract, *G:F* Gain-to-feed ratio, or feed conversion ratio, *LPS* Lipopolysaccharide, *NEFA* Free fatty acids, or non-esterified fatty acids, *OM* Organic matter, *VFA* Volatile fatty acids

Various studies have examined the effect of yeast supplementation in receiving beef steers. Finck et al. [110] conducted a study to administrate the live yeast (*S. cerevisiae*) in the receiving diet to newly weaned steers after transport with lipopolysaccharide (LPS) challenge and reported increased cumulative DMI. A recent study indicated that feeding *S. cerevisiae* to steers can improve BW, ADG and feed efficiency (gain-to-feed ratio, G:F) during receiving period (d 1 to 47) but fail to enhance cumulative growth performance during the whole receiving and backgrounding period (d 1 to 77) [111].

For finishing cattle, the supplementation of *S. cerevisiae* can improve carcass quality and total tract apparent digestibility of dry matter (DM), organic matter (OM), crude protein (CP), ether extract (EE) and fiber, but the benefit on DMI, ADG and G:F is not significant [51, 112, 113]. In contrast, Liu et al. [114] found improved ADG and ruminal propionate concentration in finishing beef cattle supplemented with live yeast, suggesting the dose of 4 g/head/d (8× 10^10^ cfu/d) of active dry yeast to have the similar carcass weight in the low plane of nutrition compared to a high nutrition plane. The inconsistent outcomes on growth may result from different sources and doses of yeast products as well as the differences in basal diets used in these trials. Recently, another study evaluated the effect of yeast supplementation on finishing cattle under heat stress and reported no beneficial effects on BW, ADG, complete blood counts, glucose, and free fatty acids, though water intake was higher and respiration rates under heat stress tended to be decreased [115]. This minimal benefit can be due to unindicated strain and live cell counts to take effects and further studies need to elucidate the biological mechanism of yeast supplementation and heat stress alleviation.

A positive impact of yeast DFM supplementation on feed intake, body weight gain and feed efficiency has also been reported for growing feedlot lambs [109], while other researchers indicated similar or reduced growth rate or growth efficiency [12, 116]. Tripathi and Karim [109] reported the improved microbial protein synthesis and feed efficiency in feedlot weaned lambs when supplemented with three live yeast cultures (*Kluyveromyces marximanus* NRRL3234, *Saccharomyces cerevisiae* NCDC42, *Saccharomyces uvarum* ATCC9080) respectively or with a mixed culture of the three (1:1:1). However, when administered with *S. cerevisiae* and *L. sporogenes* (dose: 1.5% of concentrate), lambs did not receive beneficial impact on BW, growth or carcass traits [12]. The current findings on fungal DFM supplementation indicate that their impact in growing lambs may be associated with animal diets, supplementation strains and doses.

In addition, bacterial DFMs have also been tested in receiving and feedlot ruminants. Kenney et al. [10] observed that supplementing bacterial DFMs containing *L. acidophilus* and *E. faecium* to receiving steers had no influence on their health such as morbidity and humoral immune response as well as performance (DMI, feed efficiency). Recently, bacterial DFM products have been reported to potentially reducing pathogen shedding in ruminants. Research has focused on the pathogen exclusion and DFM effect on the shedding of *E. coli* O157:H7 in feedlot beef cattle which can potentially prevent the foodborne pathogen *E. coli* transmission. *L. acidophilus* has been intensively studied on mitigating shedding of *E. coli* in finishing cattle. Wisener et al. [118] reviewed previous studies on *L. acidophilus* and concluded that the combination of *L. acidophilus* (NP51) and *P. freudenreichii* (NP24) was more effective when the supplementation dosage was higher than 10^9^ cfu/animal/d. Later, Peterson et al. [31] demonstrated that the fecal shedding of *E. coli* was reduced significantly in 448 feedlot cattle administrated with *L. acidophilus* strain NP51 in 2 years and the treated steers were 35% less likely to shed *E. coli* O157:H7 after two-year treatment. However, a recent study revealed that a combination DFM product containing *L. acidophilus* and *L. casei* cannot effectively reduce fecal shedding of *E. coli* O157:H7 in feedlot cattle [33]. It has been suggested that bacterial DFMs might be more effective in the early stage of finishing period when cattle are just introduced to the feedlot [119]. It is noticeable that once animals are adapted to bacterial DFMs, the supplementation might be less effective. This may request more complex studies evaluating how the efficacy associated with strains and dosages of bacterial DFMs in relation to the microbial adaptation. In addition, Hassan et al. [117] reported that supplementing *R. flavefaciens* to growing lambs improved digestibility, rumen fermentation and growth, and meanwhile, reduced *in vitro* methane concentrations. The multi-strain DFM consisted of LAB, LUB and yeast (*M. elsdenii* plus Lactobacilli and *S. cerevisiae*) showed advantage over other combinations (Lactobacilli, or Lactobacilli plus yeast) in improving DM digestibility, N intake, absorption and retention observed in the growing lambs, although all treated lambs had improved ADG and feed efficiency [34, 120]. This enhancement in growth can be explained by the increased microbial protein synthesis and regulated rumen microbial populations (e.g., increased fiber-degrading bacteria, lower methanogen abundance). Regardless, it is important that future research can focus on finding optimal combination of different DFM strains to achieve best outcomes in growing small ruminants.

In general, DFM supplementation to newly received beef cattle showed variability in positive outcomes according to previous studies, which may result from animal variation, difference in management of cattle prior to arriving at feedlots (animals purchased from different buyers), differences in receiving diets, or supplementation strategies (divergent DFM species or dosages, supplementation before or upon arrival) [121]. For feedlot ruminants, DFMs can be functional alternatives to promote growth, carcass quantity and quality, but the mechanisms on how different types and dosages of DFMs perform in the rumen and the lower gastrointestinal tract are still questionable. Further studies need to be conducted on selecting potential DFM products with stable and long-term effective positive outcomes. Additionally, the supplementation strategies (e.g., optimal administration period, diet types, dosages, delivery methods) also require further investigation to provide a standard for industrial implication.

#### Effect of DFMs on transition or lactation ruminants

Research about DFMs conducted on dairy ruminants has been focused on their effects on performance and health during transition and lactation periods (Table 4). Usually, dairy ruminants are at high risks during these periods due to the stress from calving, changing diets from forage-based types to high-concentrate diets, and lactation as well as negative energy balance and potential inflammations or metabolic disorders. Some studies have reported that DFMs (mainly combination of LAB, LUB, or yeast) can improve milk production, feed efficiency and health performance in dairy cows [40, 42], however, the efficacy was not consistent [122].

**Table 4:** Impact of various DFM supplementation on health and performance of transition or lactating ruminants

DFM	Dosage	Delivery method	Animal	Health	Performance	Reference
*S. cerevisiae* *E. faecium*	5 × 10^9^ cfu/d 2 × 10^9^ cfu/d	Mixed with 0.5 kg ground corn and top-dressed	Transition dairy cows	–	No effect on DMI, milk yield, BW, plasma BHBA, NEFA, glucose, haptoglobin	[24]
			Lactating dairy cows (60–70 d in milk)	–	No effect on DMI, milk yield, milk and blood parameters; lower fecal starch content, greater apparent total-tract digestibility of starch	
*S. cerevisiae*	2 × 10^10^ cfu/d	In ration	Primiparous lactating dairy cows challenged with SARA	Tended to alleviate SARA symptoms	No effect on DMI and milk yield	[122]
*S. cerevisiae*	8 × 10^10^ cfu/d	Mixed with ground corn	Multiparous lactating dairy cows	Improved ruminal pH	Increased DMI, milk yield, total VFA production, higher propionate	[40]
*S. cerevisiae* CNCM I-1077	1 × 10^10^ cfu/d	In ration	Lactating dairy cows	–	No effect on DMI, eating time, milk yield, production efficiency; tended to improve rumination, rumen temperature and milk fat production	[95]
*S. cerevisiae* CNCM I-4407	5 × 10^10^ cfu/d	Top-dressed on ration	Lactating dairy cows	Lower ruminal lactate, serum NEFA and BHBA, liver enzyme activities	Increased milk yield, rumen pH 4 h after morning feeding, total VFA and acetate concentration; No impact on propionate and butyrate concentrations; higher glucose at peak lactation	[11]
*S. cerevisiae*	4 × 10^9^ cfu/d	Incorporated into a grape by-product and mixed with basal diet	Early lactating dairy goats	Similar plasma metabolites and liver enzymes; reduced fecal *E. coli* and increased lactobacilli (greater stability of intestinal ecosystem)	Greater DMI, milk production	[123]
*S. cerevisiae*	2 × 10^10^ cfu/d	In ration	Primi- and multiparous transition and early lactating dairy sheep	Suppressed pro-inflammatory gene expression during peripartum period	Increased milk yield; tended to increase milk fat production; enhanced energy utilization	[124]
*Propionibacterium* P63 *L. plantarum* 115 *L. rhamnosus* 32	P63 or P63 + Lp or P63 + Lr (10^10^ cfu/d of each strain)	In high-starch or low-starch diet	Lactating dairy cows	–	Rumen pH increased; no effect on ruminal VFA; P63 + Lr tended to reduce CH_4_ emission with low-starch diet	[20]
*M. elsdenii*	4.8 × 10^12^ cfu/d	Inoculation through ruminal cannula for 2 d	Lactating dairy cows challenged with SARA	Increased protozoa count, decreased *S. bovis* count	Increased total VFA concentration in the corn-based group; decreased VFA concentration in the wheat-based group	[42]
*P. bryantii* 25A	2 × 10^11^ cells/dose	In ration	Dairy cows in mid-lactation challenged with SARA	No effect on SARA symptoms	No effect on rumen pH	[44]
*L. casei Zhang* *L. plantarum P-8*	1:1: 6.5 × 10^10^ cfu/d	In ration for 30 d	Primiparous lactating dairy cows	No effect on fecal bacteria richness and diversity; enhanced rumen fermentative and beneficial bacteria; suppressed potential pathogens	No effect on milk fat, protein and lactose contents; increased milk production, milk immunoglobulin G, lactoferrin, lysozyme, lactoperoxidase; decreased somatic cell counts	[125]
*S. cerevisiae* *Lactococcal*	L: 1.6 × 10^10^ cfu/d; SC + L: yeast 8 × 10^10^ cfu/d and L .8 × 10^9^ cfu/d	In ration	Healthy or mastitis lactating dairy cows	Alleviated mastitis by relieving mammary gland inflammation, reducing milk somatic cell counts, decreasing abundance of mastitis-causing pathogens (*Enterococcus* and *Streptococcus*)	–	[126]
*B. licheniformis* *B. subtilis*	2.56 × 10^9^ viable spores/d in ewe’s feed	Mixed in the corn	Late pregnancy ewes, young lambs	No significant difference in mortality (mainly due to diarrhea)	Higher milk yield for ewes and increased fat and protein content in milk	[127]
*L. reuteri DDL 19* *L. alimentarius DDL 48* *E. faecium DDE 39* *B. bifidum spp.*	10^10^ cfu/d	In ration	Mid-lactating dairy goats	More conserved morphological structures in intestine	Increased unsaturated fatty acid concentrations in milk, ruminal production of conjugated linoleic acid	[128]

*BHBA* Beta-hydroxybutyrate, *BW* Body weight, *DMI* Dry matter intake, *NEFA* Free fatty acids, or non-esterified fatty acids, *SARA* Subacute rumen acidosis, *VFA* Volatile fatty acids

The supplementation of the combination of *S. cerevisiae* and *E. faecium* (yeast: 5 × 10^9^ cfu/d; *E. faecium*: 5 × 10^9^ cfu/d) have been tested on dairy cows during the transition period. Although previous research reported positive effect on milk production and negative energy balance postpartum [21–23], when the dose of *E. faecium* changed to 2 × 10^9^ cfu/d, beneficial effect on DMI, milk yield, plasma glucose, beta-hydroxybutyrate (BHBA) or free fatty acids (NEFA) was not observed in transition cows [24]. From the same study, this DFM did not influence performance parameters in the lactation period (60–70 d in milk), but lower fecal starch content was observed, suggesting improved apparent total tract starch digestibility. The effect of the yeast *S. cerevisiae* on ruminal acidosis mitigation has been studied in dairy cows during transition and lactating periods. In primiparous lactating dairy cows fed with *S cerevisiae* (2 × 10^10^ cfu/d), although this supplementation did not improve DMI and milk yield, it showed the tendency to alleviate SARA symptoms [122]. However, multiparous lactating dairy cows responded positively to the supplementation of *S. cerevisiae* (8 × 10^10^ cfu/d) with increased DMI, milk yield, total VFA production, higher propionate, and improved ruminal pH [40]. DeVries and Chevaux [95] evaluated the effect of *S. cerevisiae* (1 × 10^10^ cfu/d) on feeding behaviors in lactating dairy cows, indicating a tendency of improving rumination, rumen mean temperature and milk fat production, although DMI, eating time, milk production had not been affected. Another study supplying a higher dose of *S. cerevisiae* (5 × 10^10^ cfu/d) to lactating dairy cows and pointed out lower liver enzyme activities, lower blood NEFA and BHBA, as well as higher glucose levels at peak lactation, suggesting the positive impact of yeast supplementation on the negative energy balance during early lactation period [11].

As another important economic sector in small ruminant industry, dairy goats are studied with *S. cerevisiae* supplementation during early lactation. Although a previous study has demonstrated that *S. cerevisiae* administration in early lactation could improve DMI and milk production in dairy goats [123], a recent study supplementing *S. cerevisiae* to dairy sheep during transition and early lactation periods reported beneficial effect on health and energy utilization but not affected milk production performance [124]. So far, it is evident that that yeast supplementation has positive influence on rumen pH regulation and energy supply in dairy ruminants, however, the previous research has not established the effective supplementation strategies (i.e., dosage, strain selection, supplementation periods) for transition and lactating stages as well as their effect on lower gut health remains unknown. As dairy ruminants are at high risk of natural immunosuppression during transition period (between late pregnancy and early lactation) due to high energy requirement but low DMI, further research is needed to understand the mechanism under the enhancement of energy supply and the suppression on pro-inflammatory gene expression in the gut or mammary gland in lactating ruminants supplemented with yeast DFMs.

As described in the previous section, in addition to fungal DFMs, bacterial DFMs have been reported to improve ruminal pH, but the effect on ruminal acidosis alleviation in dairy cows is not consistent [20, 42, 44]. Additionally, Arik et al. [42] reported increased total VFA concentration in heifers fed a corn-based diet but decreased VFA concentration in the wheat-based group, suggesting the influence of dietary grain on the efficacy of bacterial DFMs. It has also been demonstrated that the supplementation of LAB, such as *Lactobacillus spp.*, can significantly improve quality and quantity of milk production in lactating dairy cows [125] and potentially benefit mastitis control [126]. Kritas et al. [127] administered in the late pregnancy ewes with a DFM containing *B. licheniformis* and *B. subtilis* and observed higher milk yield, milk fat and milk protein. However, this supplementation did not improve mortality caused by diarrhea in the neonatal lambs. Additionally, a bacterial DFM product supplemented to mid-lactating dairy goats has shown the potential to enrich unsaturated FA concentrations in goat milk [128].

Overall, feeding DFM products consisting bacterial or fungal DFMs to dairy cattle might be efficacious for increasing production performance and health during transition and lactating periods. More research is suggested for the selection of optimal strains or doses during different stages of production for specific ruminant species.

### Challenges of application strategies of DFMs in ruminant industry

**Fig. 3:**
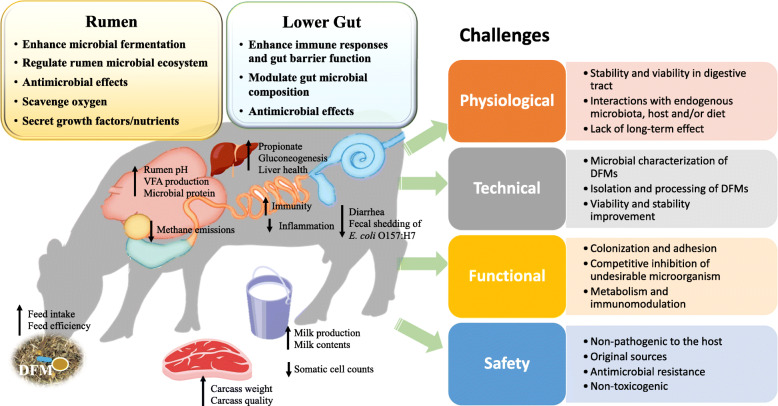
An overview of beneficial effects of DFMs on ruminants and current challenges for the development and implication of DFMs.

Although the potential beneficial impact has been identified for DFMs from many ruminant studies, there are many barriers for adoption of them to a broader context on farm (Fig. 3). In general, there are six main challenges for the implication of DFMs in ruminant industry: (1) lack of effective methods on microbial characterization of DFMs; (2) approaches to maintain the stability and viability of DFMs; (3) limited understanding of interactions of DFMs with endogenous microbiome; (4) lack of knowledge of interactions between DFMs and the host animal; (5) potential interactions of supplemented DFMs and diet; (6) lack of long-term and/or persistent effect. These barriers need to be overcome for the development of unified and effective application strategies for different ruminant species at various production stages.

#### Microbial characterization of DFMs

One of the greatest obstacles to develop the effective DFMs is to identify the microorganisms that can colonize (and become the autochthonous) and be functional *in vivo*. In probiotic studies using human and mice, it has been evident that the colonization in the gut is dependent on the strains that are derived from the genetically related host species. As the one of the first and predominant colonizers in the digestive tract of newborns [129], bifidobacteria has attracted great attention and applied as probiotics for human and animals. Their colonization factors have been reviewed and summarized extensively in the past decade [130, 131] including resistance to digestive enzymes, low pH and bile salts, carbohydrate utilization, adhesion to intestinal surface, pili, production of surface exopolysaccharide (EPS) and surface proteins. Among them, bifidobacterial EPS can influence host-bifidobacterial interaction by enhancing the adherence to the gut epithelia through the biofilm formation, modulating host immune responses, or potentially reducing colonization of the gut pathogens [132]. Moreover, the surface proteins encoded by bifidobacteria allow them to adhere to the intestine mucus (e.g., lipoprotein BopA by *B. bifidum*) and establish in the gut [133]. These factors need to be considered for selecting a candidate DFM strain. Currently, most of DFMs used in ruminants are not isolated from ruminant species. As microbes isolated from rumen environment usually failed to show benefits when administrated to ruminants, McAllister et al. [25] hypothesized that DFM strains derived from rumen may readily integrate into the microbial ecology after administration, or the cultured rumen microbes may not be able to integrate or compete within the *in vivo* rumen ecology due to their morphological and metabolic changes after repeated cultivation.

Also, potential safety risks of DFMs need to be considered regarding their virulence factors (e.g., bacteriocins) and antibiotic resistance genes (ARGs) [26]. Antibiotic resistance can naturally occur or be acquired by horizontal gene transfer [134]. The transfer of ARG of lactic acid bacteria has been well documented which may pose the risks of ARG transmission to human, especially for *Lactobacillus*, a widely used DFM in food producing animals. Given these aforementioned factors, the microbial genomics of DFMs should be performed to screen the potential functionality including CAZymes, biofilm formation, bacteriocins, antibiotic resistance genes, and other virulence factors as well as their metabolic pathways under anaerobic and/or silage production for DFM candidates to determine the most effective and safe species and strains for ruminants.

#### Stability and viability of DFMs

Adding DFM products to the diet is the easiest and practical method, and the current delivery routes are mainly orally supplemented to the ruminants by mixing with the feed or in the form of an encapsulated bolus [25]. However, the cultivation and preparation of DFMs including anaerobic microbes can be complex and prohibitive. The preparation and delivery of DFM products strictly request stability and viability in the digestive tract [29], and many factors can affect the stability and viability of the DFMs in feed and/or *in vivo*. Not all strains of the same species can survive during feed processing (e.g., heat treatment in pelleting) or in the gut, thus it is critical to ensure the viability of supplemented DFMs before feeding and after administration. The spore formation allows the bacteria resist to altered conditions during processing and storage as well as higher resistance to the intestinal environment [135]. Given this, spore-forming bacteria may have advantages as DFM candidates such as *Bacillus spp.* [29]. Additionally, nutritional and environmental factors can modulate rumen and lower gut microbiota and their activities which can in return influence the stability and viability of DFMs.

It is worth noting that the stability and viability of same DFMs are also affected by the host biological and physiological variations as well as the microbial mutation and adaptation in the gut [136, 137]. Further studies focusing on the microbial characterization of DFM strains are necessary for the selection of strains with better stability and viability in the digestive tract of ruminant. The development of monitoring and tracking methods are also needed to ensure the success colonization and effectiveness of DFMs in the gut.

#### Understanding of interactions between DFMs and endogenous microbiome

Although many studies have reported that DFM supplementation modulates the gut microbiota, the interactions between DFMs and endogenous gut microbiome is not clear, which is another key barrier for the development of effective DFMs. The recent research focused on rumen and lower gut microbiome in ruminants has enhanced our understanding of the potential interactions of DFMs and “local” microbiome. For young ruminants, it has been suggested that DFM treatment can be more effective when gut microbiota has not been fully established with mainly targeting lower gut regions [138], which helps to explain the fact that more beneficial outcomes are observed in pre-weaning animals when supplemented with DFMs. In the meantime, DFMs such as yeast has the potential capacity to promote rumen microbial microflora maturation and intestinal microbial balance in young ruminants [97, 139]. However, the understanding of gut microbiome establishment in young ruminants is still limited. Although more and more studies have revealed the taxonomic composition of rumen and lower gut microbiome in young ruminants [88, 140], the effect of DFMs on the compositional and functional changes have not been fully studied.

Compared to young ruminants, more efforts have been made to evaluate the effect of DFMs on the gut microbiome in adult animals with the focus on the rumen microbiome. As indicated in the mode of action section, bacterial DFMs can alter ruminal LAB and LUB, while yeast DFMs stimulate ruminal bacteria including fibrolytic bacteria, LUB, and amylolytic bacteria [72, 73]. In addition to rumen bacteria, the DFMs can also affect rumen methanogens [141], but it has not been revealed how supplementation of DFMs affect rumen protozoa and fungi. Compared to the rumen, the research on the effect of DFMs on lower gut microbiota is very limited. Nevertheless, most of the studies only reported the shifts in the microbial composition with little information available whether such shifts can lead to functional changes. In addition, many DFM studies have drawn the conclusion based on the comparison among treatment groups which did not take the variation in baseline endogenous microbiome into account. Recently, more and more evidence has indicated the individualized gut microbiome and such variation can significantly affect their responses and interactions with the exogenous DFMs. The metagenomic analysis have revealed the high individual ileal microbiome and functionality in preweaned calves and such variation is linked to host immune functions [142]. In addition, the research done by Maldonado-Gómez et al. showed that some individuals had longer persistence of administered *B. longum* while some did not, and the different responses were dependent on their endogenous gut microbiome [136]. Therefore, the efficacy of DFMs can be masked due to the refusal or lack of competency to stay by the endogenous gut microbiome in some individuals.

Many factors can affect the individualized endogenous microbiome in the gut of young ruminants including delivery ways, maternal factors, birth and rearing environment, first feeding strategies, management, antibiotic usage, and host factors. Among these factors, the host genetics has attracted attention, as the gut microbes have been reported to be heritable in cattle [143]. This suggests that the individual host may have different mechanisms to select who are autochthonous and who are allochthonous to establish its “preferred” gut microbiome. To date, the research investigating host genetic and physiological impact on ruminant gut microbiome as well as the mechanisms behind is still in infancy, and further understanding of the regulatory role of the host on gut microbiome is another necessary aspect for future DFM studies. Advanced technologies such as metagenomics and transcriptomics need to be applied to characterize individualized gut microbiome in ruminant species during different life stages and to investigate how DFMs establish and persist in the total digestive tract for their potential benefit impact on the host.

#### Understanding of interactions between DFMs and host animal

As aforementioned, DFMs have the capacity to interact with the host animal through direct interaction with intestinal epithelium and modulation of immune responses. There is a widely established understanding that DFMs must persist in the gut ecosystem to be effective, but the investigation on the interactions of DFMs with the intestinal cells/tissues within the complex gut environment of ruminants has just begun with the development of meta-omics techniques. Several studies have been performed to investigate DFM-host interactions, some focusing on immunomodulation effect and others on the behavior modulation through microbiota-gut-brain axis.

According to the immunomodulation effect of DFMs revealed in murine models, it has been noted that the biogeography of gut symbionts is diverse, as well as the spatial distribution of DFMs along the tract (e.g., gut lumen, mucus layers, crypts). For example, supplemented *B. adolescentis* L2–32 attached to the ileal epithelium [144] while *L. farciminis* prefers the ileum mucosa compared to the colon [145]. These suggest that the different regional gut environment (physical, chemical and biological) can directly affect the colonization of the inoculated DFMs. However, most of the previous ruminant DFM trials did not explore the host physiological and metabolic changes at molecular levels. By using germ-free models and modern molecular methods, it has been shown that the interaction of DFMs and host cells (intestinal epithelial cells and dendritic cells) occurs between host receptors and microbial ligands [146]. However, the direct impact of supplemented DFM on ruminant host cells has not been completely identified due to the lack of germ-free ruminant models. Moreover, recent research has revealed the potential roles of DFMs on host behavior and stress alleviation via the modulation through the microbiota-gut-brain axis. According to the findings in human and rodent studies, gut microbiota can influence host animal behavior by microbial metabolites and components of cell walls via various routes of interactions including the immune and enteroendocrine pathways, the enteric nervous system, and the vagus nerve [85]. Conversely, the brain can regulate the gut microbiota through modulation of gut physiology and enteric immune responses.

Overall, further research is necessary on how DFMs modulate individualized gut microbiome. Subsequently, studies on potential host gene expression changes stimulated by DFM supplementation may be required to provide us a new insight of interactions of inoculated microbes and the host. It is important to note the differences in administration doses, frequency, viability of the strains, host genotype, age, and physiological status (e.g., stress or activity) when determining the application strategies of DFMs.

#### Understanding of interactions between DFMs and diet

It is noticeable that the efficacy of DFMs can be affected by the nutritional composition and components of the diet, for example, the grain type, concentration proportion, and/or feed additives [42, 114, 147, 148]. Jeyanathan et al. [149] reported that three DFM products (*P. freudenreichii* 53-W, *L. pentosus* D31 and *L. bulgaricus* D1) failed to improve rumen fermentation in primiparous lactating dairy cows when corn silage-based high-starch diet or grass silage-based high-fiber diet was provided. In addition, other feed additives (e.g., monensin, essential oils) may impact the effect of DFMs on animal performance. Monensin, a commonly applied ionophore in North American cattle production, has been shown to alter ruminal fermentation and enhance feed efficiency through inhibition of hydrogen-producing bacteria and ammonia-producing bacteria [150, 151]. An *in vitro* study evaluated the effect of DFM supplementation on rumen fermentation of a forage-based diet in the presence and absence of monensin and revealed a tendency (*P* = 0.06) that DFM increased total VFA concentration only in the absence of monensin, suggesting the effect of the DFM-monensin interaction on total VFA concentration [147]. Koçyiğit et al. [152] suggested to feed DFM with exogenous feed enzymes to weaning and post-weaning crossbred calves to boost the beneficial effect on the growth performance. Therefore, it is essential to take the dietary composition into account to optimize the DFM supplementation strategies. Furthermore, the delivery methods of DFM products varied in different studies (e.g., in milk or starter, in powder or liquid form, top-dressed or mixed with ration), and it remains unclear whether the delivery method can affect the efficacy and what delivery method is the best or optimal for which DFM products. Further research is needed to define the effective supplementation delivery methods for different DFM types and animal production stages.

#### Lack of long-term effect

To date, not many studies have tested the long-term effect of DFM supplementation in livestock species. The short-term effect can be associated with DFMs’ capacity of persistency and colonization in the gut of adult ruminants. Some recent studies have demonstrated the potential to manipulate rumen microbiota through early life dietary interventions to receive long-term impact (e.g., enteric methane emission mitigation) [153, 154]. Therefore, DFM supplementation in the early-life young ruminants may give us a potential opportunity to obtain persistent benefit, but further studies are required in this field. Another possible reason might be the association with animal diet. Since diets can change ruminal microbial ecology [155], the shifts in diets during different periods of animal farming may result in the lack of long-term effect of DFM supplementation or request repeated supplementation. Another assumption might be the specific selection of host-adapted strains in ruminants. Moreover, the above addressed host and endogenous microbiome factors can affect the long-term effect of DFM supplementation. To address this barrier, long-term *in vitro* or *in vivo* trials are necessary to include the stability and viability, interactions with host and microbiome, and the functional changes of host and gut microbiota in ruminants.

### Concluding remarks and future perspectives

In summary, it is evident that feeding bacterial and/or fungal DFMs have significant potential to decrease diarrhea in newborn ruminants, relieve stress response in weaning, receiving and calving, increase milk production in lactating ruminants, and enhance growth in growing and finishing animals. In addition, some DFMs showed beneficial effect on methane mitigation and reduction of fecal shedding of *E. coli* O157:H7 in feedlot ruminants. Both bacterial and fungal DFMs have been shown to function regulating rumen fermentation, ruminal pH, and microbial ecosystem in the rumen. Some bacterial species or combinations are reported to increase propionate production, energy utilization, improve feed efficiency and mitigate enteric methane emissions. Among all, yeast products are intensively studied and currently widely applied in the livestock industry because of their functional and cost-effective characteristics. However, the modes of action of proposed yeast species have not been clearly understood, especially in the lower gut of ruminant species. And the commercial yeast products usually contain not only one strain but may combine with other substances (e.g., culture extract, cell wall, bacterial DFM strains). A comprehensive understanding of the mode of action and potential interactions of yeast and bacterial DFM strains in a combined product would promote the development of effective yeast products in the market as well as the implication. Different from fungal DFMs, some concerns arise regarding the application of bacterial DFMs. With the presence of bacteriocins and the existing reported resistance issue to bacteriocins, the future development of bacterial DFMs and their application may face obstacles and pressure from regulatory issues.

Overall, selecting effective DFM supplementation strategies to different species of ruminants from various production systems is still challenging due to the lack of knowledge in the microbial characterization of DFM products, their stability and viability in storage and within the gastrointestinal tract, their interactions with the endogenous microbiota and the host, as well as the potential long-term effect on animal health and production. Therefore, it is suggested to apply the advanced technologies in system biology to reveal the mode of action of strains that could be selected as potential DFMs, as well as the host-microbe, microbe-microbe interactions. At the meantime, investigating the potential negative effects of microorganisms in terms of antimicrobial compound excretion and antimicrobial resistance can boost the future development of DFMs. The host, diet and environmental factors should also be considered for the optimal DFM application strategies.

## Data Availability

Not applicable.

## Abbreviations

ADG: Average daily gain
ARGs: Antibiotic resistance genes
BHBA: Beta-hydroxybutyrate
BRD: Bovine respiratory disease
BW: Body weight
CP: Crude protein
DFMs: Direct-fed microbials
DM: Dry matter
DMI: Dry matter intake
EE: Ether extract
EPS: Exopolysaccharide
FAO: Food and Agriculture Organization of the United Nations
G:F: Gain-to-feed ratio, or feed conversion ratio
GIT: Gastrointestinal tract
IgA: Immunoglobulines A
LAB: Lactic acid producing bacteria
LPS: Lipopolysaccharide
LUB: Lactic acid utilizing bacteria
NEFA: Free fatty acids, or non-esterified fatty acids
OM: Organic matter
SARA: Subacute ruminal acidosis
VFA: Volatile fatty acids
WHO: World Health Organization
